# Impact of the Inflow Population From Outbreak Areas on the COVID-19 Epidemic in Yunnan Province and the Recommended Control Measures: A Preliminary Study

**DOI:** 10.3389/fpubh.2020.609974

**Published:** 2020-12-02

**Authors:** Zhong Sun, Guozhong He, Ninghao Huang, Hongyu Chen, Shuwei Zhang, Zizhao Zhao, Yao Zhao, Guang Yang, Songwang Yang, Haiyan Xiong, Thilakavathy Karuppiah, S. Suresh Kumar, Jibo He, Chenglong Xiong

**Affiliations:** ^1^Department of Biomedical Science, Faculty of Medicine & Health Sciences, Universiti Putra Malaysia, Serdang, Malaysia; ^2^School of Public Health, Kunming Medical University, Kunming, China; ^3^Department of Epidemiology, School of Public Health, Fudan University, Shanghai, China; ^4^Key Laboratory of Public Health Safety, Ministry of Education, School of Public Health, Fudan University, Shanghai, China; ^5^Genetics and Regenerative Medicine Research Group, Faculty of Medicine & Health Sciences, Universiti Putra Malaysia, Serdang, Malaysia; ^6^Department of Medical Microbiology and Parasitology, Universiti Putra Malaysia, Serdang, Malaysia; ^7^Center for Disease Control and Prevention in Yunnan Province, Kunming, China

**Keywords:** COVID-19, control measures, epidemic area, resettlement, inbound travelers

## Abstract

**Background:** COVID-19 developed into a global pandemic in 2020 and poses challenges regarding the prevention and control capabilities of countries. A large number of inbound travelers from other regions could lead to a renewed outbreak of COVID-19 in the local regions. Globally, as a result of the imbalance in the control of the epidemic, all countries are facing the risk of a renewed COVID-19 outbreak brought about by travelers from epidemic areas. Therefore, studies on a proper management of the inbound travelers are urgent.

**Methods:** We collected a total of 4,733,414 inbound travelers and 174 COVID-19 diagnosed patients in Yunnan province from 21 January 2020 to 20 February 2020. Data on place of origin, travel history, age, and gender, as well as whether they had suspected clinical manifestations for inbound travelers in Yunnan were collected. The impact of inbound travelers on the local epidemic was analyzed with a collinear statistical analysis and the effect of the control measures on the epidemic was evaluated with a sophisticated modeling approach.

**Results:** Of the 174 COVID-19 patients, 60.9% were not from Yunnan, and 76.4% had a history of travel in Hubei. The amount of new daily cases in Yunnan was significant correlated with the number of inbound travelers from Hubei and suspected cases among them. Using Susceptible–Exposed–Infectious–Recovered (SEIR) model analysis, we found that the prevention and control measures dropped the local R0 down to 1.07 in Yunnan province.

**Conclusions:** Our preliminary analysis showed that the proper management of inbound travelers from outbreak areas has a significantly positive effect on the prevention and control of the virus. In the process of resettlement, some effective measures taken by Yunnan province may provide an important reference for preventing the renewed COVID-19 outbreak in other regions.

## Introduction

COVID-19, a novel coronavirus disease that appeared in late 2019, has spread to the majority of countries worldwide, and has resulted in a substantial number of deaths. It was also named SARS-CoV-2 because its symptoms are similar to those of severe acute respiratory syndrome (SARS). COVID-19 was defined as a Public Health Emergency of International Concern by the World Health Organization (WHO) on 30 January 2020 (1). Advances in transportation technology have made life easier and more convenient, and according to data from various destinations, international arrivals (overnight visitors) reached 1.5 billion in 2019 (2). Concurrently, these conveniences have also accelerated the rate of spread of viruses. As international travel increases, travelers are more able to spread infectious diseases acquired in their home countries to their destination, as well as being able to spread diseases from their destination to their home countries. Because of the aforementioned reasons, COVID-19 constituted a major challenge to international public health. According to the prediction model, the basic reproduction number (R0) of COVID-19 is about 2.68 (3). As of 11 March 2020, COVID-19 had already erupted in more than 110 countries, with more than 118,000 confirmed cases, and 4,291 people having lost their lives. On the same day, the World Health Organization (WHO) declared the COVID-19 outbreak a global pandemic (4).

Since the outbreak of COVID-19, in response to the spread of such viruses through travel, many countries have implemented large-scale blockades to slow the spread. For example, an unprecedented lockdown was imposed by the Chinese government in Wuhan from 23 January 2020, with travel restrictions. Within a few days, the quarantine expanded to other provinces and cities, affecting more than 50 million people in total (5). Unfortunately, more than 5 million people left Wuhan before lockdown due to the upcoming Spring Festival, many of whom may have been infected with the virus (6). Thus, these travelers who left the affected area before the blockade were able to spread COVID-19 to other parts of China and around the world. Although countries have adopted active countermeasures to prevent both imported and exported cases, most countries in the world will be faced with undetected travelers from affected areas moving to non-affected areas.

Yunnan province, a very popular tourist destination, ranking third in China, received a total of 7.0608 million domestic and foreign overnight tourists in 2018 (7). Most provinces in China refused to accept tourists from Hubei during the epidemic. However, Yunnan province became a gathering place for travelers from Hubei, since it allowed entry to Hubei tourists. In this preliminary study, we therefore selected Yunnan Province as an example to discuss the management of travelers from affected areas after an outbreak. To evaluate the effectiveness of the measures taken in Yunnan, we collected and analyzed the monitoring data of inbound travelers and the isolation data of hotels during the outbreak in Yunnan Province. The results of this study may provide some reference and guidance on how to regulate outbound travelers from affected areas in the future.

## Materials and Methods

### Epidemiological Data

We survey all 174 patients diagnosed as COVID-19 in Yunnan province. The first COVID-19 patient in Yunnan Province was diagnosed on 21 January 2020, and the 174th patient was diagnosed on 20 February 2020, with no new cases within the next 2 weeks. All the data were collected from the Epidemic Command Center, which is composed of the People's Government of Yunnan Province, the Health Commission of Yunnan Province, the Yunnan Center for Disease Control and Prevention, the Yunnan Provincial Department of Culture and Tourism, and the Department of transport of Yunnan Province. The province-wide control of Yunnan province began on 28 January 2020. The data include surveys of the inbound travelers in eight Prefectural-level municipalities and eight Autonomous prefectures in Yunnan province from 28 January to 20 February 2020. The content of the investigation includes daily visitors to Yunnan, who were asked their domicile of origin and whether they had suspicious clinical manifestations (such as an abnormal body temperature, cough, shortness of breath, etc.). In addition, it also includes the number of new daily cases, deaths, and recovered cases during this period. We also extracted data on gender, age, place of origin, and history of travel in Hubei of all 174 patients during this period. The data used are officially released, all patients are anonymous, and do not involve any personal privacy, therefore, no ethical approval is required from author corporate for the people and animals.

### Data Analysis

All data were proofread and double entered using EpiData3.1 software to ensure accuracy. Data were transformed into the database through Excel 2019 software, and statistically analyzed using SPSS version 25.0. Based on patients' place of origin, we categorized the patients into three groups (e.g., Hubei, Yunnan, other provinces). To analyze the differences on gender, age, and history of travel in Hubei among these three different groups, a descriptive statistical analysis was conducted and Chi-squire tests were performed for these 174 diagnosed patients. In addition, multiple linear regression analysis was used to screen for variables using stepwise methods. A final model was constructed with the independent variable of the number of new cases per day in Yunnan Province, and the dependent variables of the amount of daily inbound travelers (from Hubei Province) and the amount of suspected infected persons among them. A *p*-value of <0.05 (typically <0.05) was considered statistically significant.

Furthermore, we parameterized the above data (also including the daily deaths and recoveries in Yunnan) based on the Susceptible–Exposed–Infectious–Recovered (SEIR) model (8) and estimated the R0 (basic reproduction number of the disease transmission) under the control interventions of Yunnan with Matlab software (MATrix LABoratory) (9).

## Results

### Basic Characteristics of COVID-19 Cases in Yunnan Province

**Figure 1:**
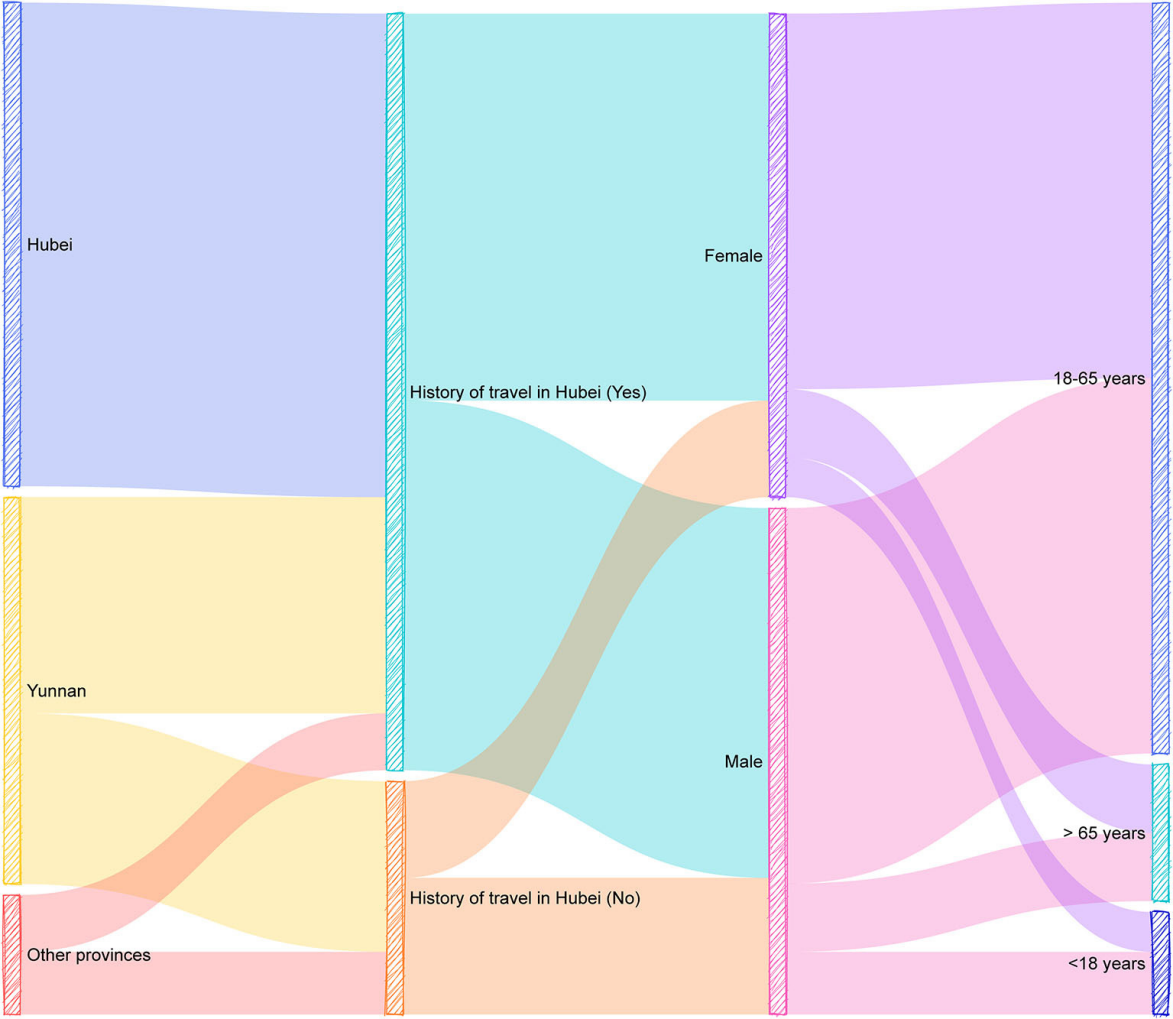
Illustration of descriptive statistical characteristics of 174 COVID-19 patients.

From the first suspected case being confirmed as a pneumonia case caused by SARS-CoV-2 on 16 January 2020, Yunnan Province accumulated 174 confirmed cases as of 24 February. Table 1 and Figure 1 show the results of the descriptive statistical analysis of 174 patients.

**Table 1:** Descriptive statistical characteristics of 174 COVID-19 patients (place of origin, history of travel in Hubei, gender, and age).

	**Place of Origin**				
**Characteristics**	**Hubei (*n* = 85)**	**Yunnan (*n* = 68)**	**Other provinces (*n* = 21)**	**Chi-square**	***P*-value**
History of Travel in Hubei				51.836	0.000
Yes	85 (100.00)	38 (55.88)	10 (47.62)		
No	0	30 (44.12)	11 (52.38)		
Sex				0.203	0.904
Male	42 (49.41)	36 (52.94)	11 (52.38)		
Female	43 (50.59)	32 (47.06)	10 (47.62)		
Age Distribution				9.485	0.050
<18 years	9 (10.59)	9 (13.24)	1 (4.76)		
18–65 years	60 (70.59)	54 (79.41)	20 (95.24)		
>65 years	16 (18.82)	5 (7.35)	0		

Among the 174 confirmed COVID-19 cases, there were 85 cases originally from Hubei province (48.9%), 68 cases originally from Yunnan province (39.1%) and 21 cases originally from other provinces (12.1%), indicating that majority of the patients (70.9%) did not originally come from Yunnan. Regarding the travel-history in Hubei, A total of 133 cases (76.4%) had a history travel in Hubei province. The number of male patients was slightly higher than that of female patients [89 males (51.1%) and 85 females (48.9%)]. In addition, patients were mainly distributed in the 18–65 years group (132 cases, 75.9%). The youngest patient was 3 years old, and the oldest patient was 83 years old. The average age of the patients was 41 years. Among 174 COVID-19 patients in Yunnan province, only two patients died, and both of whom were males older than 65 years. The other 172 patients were recovered by the end of this study.

### Analysis of the Correlation Between the Daily Number of New Cases and the Inbound Population

Table 2 shows the daily arrivals in Yunnan province and suspected infected persons among them from 28 January 2020 to 20 February 2020, and the daily arrivals in Yunnan province from Hubei and the suspected infected persons among them. Table 2 also lists the new daily cases of COVID-19 in Yunnan Province during this period. The amount of daily inbound travelers and the number of suspected infected persons among them are listed separately. In addition, we provided the information on the amount of daily inbound travelers from Hubei and the number of suspected infected persons among them separately.

**Table 2:** List of daily inbound travelers and new daily cases, recovered, and deaths in Yunnan from 28 January 2020 to 20 February 2020.

**Date**	**Inbound Travelers from Hubei**		**Inbound Travelers**		**New Daily Cases**	**New Daily Recovered**	**New Daily Deaths**
	**Total**	**Suspected Cases**	**Total**	**Suspected Cases**			
1.28	17,857	438	143,469	860	25	0	0
1.29	23,141	480	238,092	722	19	0	0
1.30	27,598	598	233,520	1,209	10	0	0
1.31	29,201	570	254,982	1,176	11	1	0
2.1	39,884	568	212,555	1,501	8	1	0
2.2	36,634	522	316,851	1,067	10	1	0
2.3	38,641	524	257,567	808	8	2	0
2.4	35,688	460	258,137	619	5	0	0
2.5	35,530	457	266,758	691	6	0	0
2.6	36,936	462	267,514	753	7	2	0
2.7	36,324	444	272,907	941	3	5	0
2.8	35,495	459	278,214	733	2	5	0
2.9	36,410	435	284,578	813	1	1	0
2.10	34,813	437	306,706	790	8	1	0
2.11	29,450	192	162,175	597	5	1	0
2.12	26,130	159	139,795	523	1	3	0
2.13	20,037	95	106,101	445	7	4	0
2.14	19,713	96	116,493	338	6	8	0
2.15	18,900	90	105,613	349	1	7	0
2.16	17,523	88	102,758	304	2	0	0
2.17	17,017	114	102,592	258	1	5	0
2.18	16,786	85	102,036	220	1	10	0
2.19	16,495	74	101,310	209	0	3	1
2.20	15,370	61	102,691	191	1	19	1
Total	661,573	7908	4,733,414	16,117	148	79	2

With the multiple linear regression analysis, we found that the number of inbound travelers and suspected cases among them are not significantly correlated with the number of new daily cases, and the *p*-values are 0.730 and 0.879, respectively. However, the number of inbound travelers from Hubei and suspected cases among them are significantly correlated with the number of new daily cases with the *p*-values of 0.018 and 0.024, respectively.

**Figure 2:**
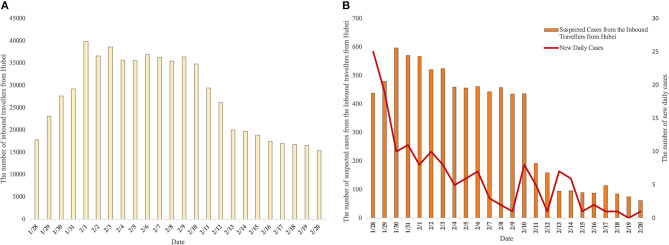
Time series chart of variables related to new daily cases in Yunnan Province. **(A)** Shows the number of daily inbound travelers from Hubei. **(B)** Shows the number of suspected cases from inbound travelers from Hubei and new daily cases.

The stepwise method was used to eliminate the two independent variables: the number of inbound travelers and suspected cases among them. A linear regression model can be obtained with R square = 0.634, F = 18.203, and a *p*-value of <0.05. The model is as follows: the number of new cases per day = 11.42 – 0.000655^*^ the number of inbound travelers from Hubei + 0.03886^*^ suspected cases from the inbound travelers from Hubei. Meanwhile, we combined the three variables in the model with time. The time series plot is shown in Figure 2.

### Epidemic Model

**Figure 3:**
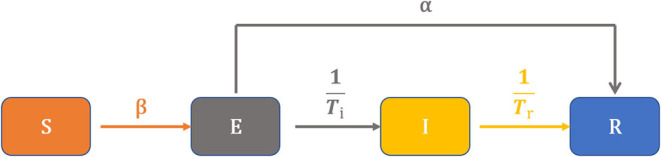
Diagram of the model used in this study for simulating the COVID-19 infection.

Based on the daily number of new cases, recovered cases, and deaths in Yunnan, we constructed the SEIR model (Figure 3) to describe the status of each compartment shown in the following differential equations:

$$\begin{array}{l} N=S+E+I+R\\ \frac{dS}{dt}=-\beta I\frac{S}{N}\\ \frac{dE}{dt}=\beta I\frac{S}{N}-\frac{1}{T_{i}}E-\alpha E\\ \frac{dI}{dt}=\frac{1}{T_{i}}E-\frac{1}{T_{r}}I\\ \frac{dR}{dt}=\frac{1}{T_{r}}I+\alpha E \end{array}$$

We stratified the populations into susceptible (S), exposed (E), infected (I), and recovered or removed (R). Furthermore, N is the total number of people, β is disease transmission rate, α is the rate of (I) direct conversion to (R). *T*_*i*_ is days of (E) converted to (I), and *T*_*r*_ is days of (I) converted to (R).

We fitted the data according to the above model. Through data simulation, the number of infected people was calculated by the fitting prediction. Among them, the loss function is the mean variance of the actual number of infected people minus the predicted number of infected people. The minimum value of the loss function was found by using the gradient descent method. With the Matlab software (MATrix LABoratory), we concluded that

$$\begin{array}{l} R0=({T_{I}}^{2}\times {T_{r}}^{2}\times \alpha^{2}-2\times {T_{i}}^{2}\times T_{r}\times \alpha +{T_{i}}^{2}+2\times T_{i}\\ \;\times {T_{r}}^{2}\times \alpha +4\times \beta \times T_{i}\times {T_{r}}^{2}-2\times T_{i}\times T_{r}+{T_{r}}^{2}{)}^{\frac{1}{2}}.\\ \;÷\left(T_{i}\times T_{r}\times \alpha +T_{i}+T_{r}\right) \end{array}$$

**Figure 4:**
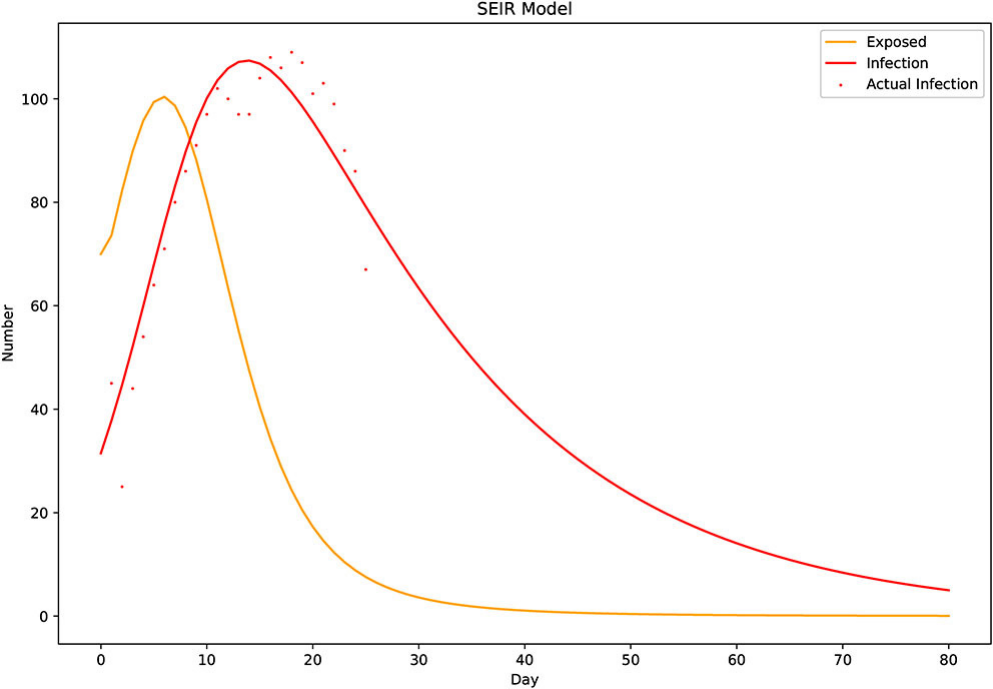
The Susceptible–Exposed–Infectious–Recovered (SEIR) model. A simulation graph drawn according to the model and the number of known daily infections, deaths, and recoveries. According to the model, epidemics occur because R0 > 1, although the fit is not optimal at the beginning of the epidemic. The calculations indicate that unsureness is related to the initial number of exposed individuals. The incubation period *T*_*i*_ is 8.6 days, which can be reflected in the SEIR model (Figure 4). The infection curve (red) is one incubation period later than the exposure curve (yellow), and the gap continues to widen in the later stages. Because the rate of progression from exposure to infectious (1/*T*_*i*_) was greater than the recovery rate of infectious individuals (1/*T*_*r*_), the number of infected people declined faster than the number of exposed people.

For this model, there is no endemic equilibrium as the S, E, I, R in the model will all return to zero once the epidemic has finished. We linearized our system around each equilibrium using the Jacobian matrix evaluated at the chosen equilibrium. Otherwise, the local equilibrium was unstable (10). Based on this equilibrium, we obtained the values of this area: β = 11.12, α = 5.11, *T*_*i*_ = 8.64, *T*_*r*_ = 18.84, *R*0 = 1.07 (Figure 4).

## Discussion

Although Hubei province implemented a strict city closure order, there were still many people from Hubei moving outside, which is what we mean by inbound travelers from affected areas. Meanwhile, Yunnan Province became the first province in China to release information about designated hotels during the outbreak. As of 17 February 2020, the number of designated resettlement hotels increased to 187, and the cumulative number of tourists resettled in Yunnan reached 42,493, including 5,816 tourists from Hubei and 2,552 from Wuhan (11). However, it is worth noting that under the strict prevention and control measures implemented in Yunnan, COVID-19 did not break out locally, and our study suggested that the local R0 dropped to 1.07 in Yunnan Province is worthy of reference and discussion.

SARS-COV-2 (R0 = 2.68) seems to be more contagious than SARS-CoV (R0 = 2.2) and MERS-CoV (R0 = 1.21) (3, 12, 13). The statistics from 174 patients with COVID-19 contained 89 males, accounting for about 51.1%, slightly more than females. Majority of patients were in the 18–65 age group, accounting for ~75.9%, and the median age was 40 years. Furthermore, only two COVID-19 patients died in Yunnan province, both of whom were males older than 65 years. The incidence of male morbidity is higher than that of females, which has also been confirmed in two other studies (14, 15). This may be related to females having a higher innate and adaptive immune response than men, leading to faster elimination of the virus (16). However, the corresponding pathogenic and molecular biological foundations for this still need to be explored further.

In the place-of-origin statistics from the 174 patients, we found that non-Yunnan patients accounted for 60.9%. Meanwhile, in the history-of-travel statistical results of all the patients, people from or who had been through Hubei accounted for 76.4% of the total number. All of the above indicates that the diagnosed patients in Yunnan Province were closely related to the outflow population from Hubei Province. During the outbreak of COVID-19 in China, we became aware of the potential insecurity factor. As a result of the large-scale blockades and control activities in China, a lot of outbound travelers from epidemic areas became homeless. Meanwhile, they were often unable to enter Hubei directly and were quarantined in other cities for 2 weeks, after which most of them were required to return to their places of origin. However, as a result of the epidemic in Hubei and the closure of the city, some outbound travelers from Hubei did not want to return to the epidemic areas. Because of their specific situation, people in most cities did not want to accept them. Even when a small number of outbound travelers intended to return to the affected areas, they were refused admittance to public/private transport facilities due to their identity and destination. As a result of the above factors, there was a phenomenon in which outbound tourists could not return home and thus became homeless.

In our statistical study, the above phenomenon has been confirmed. Even though the authorities adopted a strict city closure directive, there were still numerous people from the epidemic areas entering Yunnan province every day. Meanwhile, our correlation analysis also confirmed that the new daily cases of COVID-19 in Yunnan had a significant correlation with the number of inbound travelers from Hubei and the suspected cases among them. This shows that during the outbreak, the outflow of people from affected areas is not only the main reason for the persistence and spread of the epidemic, but also the main source of imported cases overall.

In this study, according to the model we established, the management and control of outbound travelers from epidemic areas can effectively and rapidly control the spread of the epidemic. Therefore, this study also focuses on investigating the measures taken to quickly control the current epidemic in Yunnan Province. We believe that the following prevention and control measures implemented in Yunnan are worthy of reference and promotion.

First, in the prevention and control of the epidemic, all inbound travelers should be screened and checked, with special attention being paid to those who have traveled from affected areas. In China, the Department of Telecommunications provides mobile phone users with free travel history inquiry services, which can be provided to the relevant infection prevention and control department to facilitate the registration and verification of whether the user has been to an epidemic area (17). As human beings enter the information age, although the speed of virus transmission is much faster than before, big data can assist human beings in the prevention and control of infectious diseases to a certain extent (18).

Second, the lower floors of designated hotels should be used for those who need be separated. As early as 2003, when the SARS virus broke out in Hong Kong, researchers found that people on the lower floors were infected by the virus by people on the upper floors who were infected and also had symptoms of diarrhea. The virus can spread as an aerosol through a sewer pipe to the bathroom system downstairs through feces. This way, resident's downstairs can become infected by coming into contact with small droplets containing the virus (19). In a study of SARS-CoV-2, although the patient had no symptoms of diarrhea, samples from the toilet and sink in their room were positive, indicating that virus shedding in feces may be a potential route of transmission (20). Therefore, arranging people who need attention on the lower floors is helpful to reduce the potential aerosol infection caused by the drainage system.

Third, positive and effective countermeasures should be taken to deal with the extensive environmental pollution that may be caused by tenants. Although COVID-19 patients cause extensive environmental pollution, a study showed that the air samples were negative since the air in isolation chambers is exchanged 12 times per hour, though the swab test of the exhaust port was positive (20). The data suggested that increased air circulation is conducive to virus dilution. It also proves that the air loaded with virus aerosol is discharged through the exhaust device, with some small water droplets carrying virus being deposited on the vent and other devices during the air discharge process (20). Therefore, it is necessary to maintain indoor air circulation. Each floor and each room need to improve ventilation by opening windows (usually for more than 3 h). At the same time, exhaust devices such as exhaust fans can be turned on to enhance indoor air flow. In places where centralized air-conditioning is used, the return air must be closed and the air conditioning filter must be cleaned regularly. A survival study on SARS-CoV-2 showed that it can survive on metal, glass, and plastic surfaces for anywhere from 2 h to 9 days (21). It has also been reported that alcohol-containing disinfectants can effectively kill the viruses attached to the surface of objects (21). Therefore, the indoor floors, walls, public supplies of guest rooms should be disinfected once a day. Moreover, elevator buttons, doorknobs, and other frequently touched parts should be disinfected at least three times a day.

While in various countries where the epidemic has been brought under control, there is still a potential risk of recurrent outbreaks due to travel between affected and non-affected areas (22). As early as 2016, a sample survey of nasal and pharyngeal swabs of foreign tourists without respiratory symptoms in New York City found that 6.2% of travelers tested positive for respiratory viruses, of which 38.7% carried coronavirus (23). In a survey on SARS-CoV-2, it was found that the results of nasal and pharyngeal swab tests in asymptomatic or mild-symptom patients showed similar virus levels to those of symptomatic patients, indicating that asymptomatic patients were also capable of transmission (24). Therefore, when a person is in close contact with someone who has no respiratory symptoms (within 1 m), their mucous membranes (mouth and nose) or conjunctiva (eyes) are also at risk of being infiltrated by droplets. Therefore, it is necessary to wear a mask when contacting people from affected areas (25).

## Conclusion

With the unsynchronized development of COVID-19, countries and regions will face various forms of imported cases. How to manage these imported cases is the top priority. Because it was a province that accepted people from affected areas during the outbreak of the epidemic in China for a long period of time, Yunnan province applied some temporary and effective public health interventions to prevent the virus from breaking out in the region. Although only a small number of cases included in this preliminary study, the results demonstrated that positive screening of inbound travelers and proper resettlement of travelers from epidemic areas have a very positive effect on the prevention and control of the outbreak. In the process of accommodating people from infected areas, we believe that they should be placed on the lower floors, and all hotel personnel should wear masks before coming into contact with them. Meanwhile, maintaining hand hygiene, a safe social distance, and using alcohol-based disinfectants can effectively reduce the spread of the virus. These effective prevention and control measures may provide some reference for preventing the renewed COVID-19 outbreak in other regions.

## Data Availability Statement

The original contributions presented in the study are included in the article/supplementary materials, further inquiries can be directed to the corresponding author/s.

## Author Contributions

ZS designed, drafted, and edited the manuscript. JH provided data and edited the manuscript. NH edited the figures. HC, SZ, ZZ, YZ, GY, and SY collected data. HX and SK edited the manuscript. TK reviewed and edited the manuscript. GH and CX conceptualized and designed framework of manuscript. All authors have made contribution to this manuscript and have read and agreed to the published version of the manuscript.

## Conflict of Interest

The authors declare that the research was conducted in the absence of any commercial or financial relationships that could be construed as a potential conflict of interest.
